# Recombinant Human Thrombopoietin Treatment Promotes Hematopoiesis Recovery in Patients with Severe Aplastic Anemia Receiving Immunosuppressive Therapy

**DOI:** 10.1155/2015/597293

**Published:** 2015-03-11

**Authors:** Huaquan Wang, Qi'e Dong, Rong Fu, Wen Qu, Erbao Ruan, Guojin Wang, Hong Liu, Yuhong Wu, Jia Song, Limin Xing, Jing Guan, Lijuan Li, Zonghong Shao

**Affiliations:** Department of Hematology, General Hospital, Tianjin Medical University, Tianjin 300052, China

## Abstract

*Objective*. To assess the effectiveness of recombinant human thrombopoietin (rhTPO) in severe aplastic anemia (SAA) patients receiving immunosuppressive therapy (IST). *Methods*. Eighty-eight SAA patients receiving IST from January 2007 to December 2012 were included in this retrospective analysis. Of these, 40 subjects received rhTPO treatment (15000 U, subcutaneously, three times a week). rhTPO treatment was discontinued when the platelet count returned to normal range. Hematologic response, bone marrow megakaryocyte recovery, and time to transfusion independence were compared. *Results*. Hematologic response was achieved in 42.5%, 62.5%, and 67.5% of patients receiving rhTPO and 22.9%, 41.6%, and 47.9% of patients not receiving rhTPO at 3, 6, and 9 months after treatment, respectively (*P* = 0.0665, *P* = 0.0579, and *P* = 0.0847, resp.). Subjects receiving rhTPO presented an elevated number of megakaryocytes at 3, 6, and 9 months when compared with those without treatment (*P* = 0.025, *P* = 0.021, and *P* = 0.011, resp.). The time to platelet and red blood cell transfusion independence was shorter in patients who received rhTPO than in those without rhTPO treatment. Overall survival rate presented no differences between the two groups. *Conclusion*. rhTPO could improve hematologic response and promote bone marrow recovery in SAA patients receiving IST.

## 1. Introduction

Aplastic anemia (AA) is a bone marrow failure syndrome characterized by pancytopenia and hypocellular marrow in the absence of abnormal infiltration and reticulin proliferation. Severe aplastic anemia (SAA) is often fatal if untreated. Acquired SAA is an immune-mediated disorder, with destruction of hematopoietic stem cells and progenitor cells by active T lymphocytes. Allogeneic hematopoietic stem cell transplantation (allo-HSCT) from an HLA-identical sibling donor, together with immunosuppressive therapy (IST), is the first-line treatment for SAA patients. In the absence of an HLA-identical donor or in patients older than 40 years, the use of IST in combination with antithymocyte globulin (ATG), antilymphocyte globulin (ALG), and cyclosporine is the recommended choice for SAA treatment, with a hematologic response rate of 60–75% and long-term survival rate of 70–80%. If newly diagnosed SAA patients are younger than 40 years and have a suitable sibling donor, allo-HSCT is the initial treatment [1]. Conversely, graft-versus-host disease, graft failure, and infections are common complications associated with a reduced remission rate, especially in older patients. However, about one-third of refractory SAA patients who have no suitable donor for HSCT continue to develop severe cytopenia and are at high risk for life-threatening hemorrhage as a result of thrombocytopenia and severe infections as a result of neutropenia.

Thrombopoietin (TPO) is the principal endogenous regulator of platelet production through binding of the TPO receptor, c-MPL. TPO stimulates the proliferation and differentiation of megakaryocytes, leading to increased platelet production. Recombinant human thrombopoietin (rhTPO) administration has been demonstrated to increase platelet count in patients with immune thrombocytopenia, chemotherapy-induced thrombocytopenia, myelodysplastic syndromes (MDS), and platelet apheresis donors [2–5]. However, rhTPO treatment was rarely used because of the increased serum TPO levels in AA patients. Recently, several studies have indicated that rhTPO therapy may help recover hematopoiesis in AA patients. In this line, some studies demonstrated that c-MPL is expressed in hematopoietic tissues including hematopoietic stem cells, progenitor cells, and megakaryocyte colony forming cells, suggesting a potential therapeutic role for TPO receptor agonists in AA [6, 7].

The purpose of this study was to retrospectively explore the effectiveness of rhTPO administration in SAA patients receiving IST.

## 2. Cases and Methods

### 2.1. Patients

This study included a cohort of 88 adult patients who were diagnosed with SAA according to the standard criteria [8]. SAA was diagnosed if at least two of the following criteria were met: (i) neutrophil count less than 0.5 × 10^9^/L, (ii) platelet count less than 20 × 10^9^/L, and (iii) reticulocyte count less than 20 × 10^9^/L with a hypocellular bone marrow (cellularity of less than 25%). Very severe aplastic anemia was diagnosed if the above criteria for SAA were fulfilled and the neutrophil count was less than 0.2 × 10^9^/L. Patients presenting with congenital AA were excluded from the study. Patients were screened for paroxysmal nocturnal hemoglobinuria using anti-CD55 and anti-CD59 antibodies using flow cytometry. All patients presented normal bone marrow cytogenetic values. From January 2007 to December 2012, all patients were treated with standard IST: antilymphocyte globulin (ALG, Genzyme Polyclonals S.A.S., France; 5 mg/kg/d for 5 consecutive days, intravenous) and cyclosporine (CsA; plasma concentration at 200 to 400 ng/mL, for at least one year). Patients who received allo-HSCT or the second course of ATG were excluded from this study. The present study was approved by the Ethics Committee of the Tianjin Medical University General Hospital. All patients or their legal guardians signed written informed consent in accordance with the Declaration of Helsinki.

### 2.2. Therapy

Between January 2010 and December 2012, upon completion of ATG therapy, a cohort of 40 patients (18 men and 22 women, with a median age of 36) received a subcutaneous injection of rhTPO (15000 U, 3 times a week, Sansheng, China) for an average of 7.8 months (ranging from 1.1 to 34 months). The therapeutic dose was chosen owing to the pharmacokinetics and long-term safety of rhTPO. rhTPO treatment was discontinued when the platelet count returned to the normal range (100–300 × 10^9^/L). A control group included 48 patients (27 men and 21 women, with a median age of 32) without rhTPO treatment that was selected based on the same criteria (including IST and supportive cares) between January 2007 and December 2009. Patients received platelet transfusions for a platelet count less than 10 × 10^9^/L and less than 20 × 10^9^/L with bleeding and/or fever. Patients received packed red blood cell transfusions at hemoglobin levels of <70 g/L. Bone marrow aspiration and biopsy was carried out every 3 months for a period of 9 months after the completion of IST. Responses to treatment were defined as previously reported [7]. Hematological response was defined as no longer meeting criteria for SAA without transfusion or granulocyte colony stimulating factor (G-CSF). Complete response (CR) was defined as satisfaction of all three peripheral blood count criteria: (i) hemoglobin ≥110 g/L, (ii) neutrophil count ≥1.5 × 10^9^/L, and (iii) platelet count ≥100 × 10^9^/L. Partial response (PR) was defined as improved blood counts but no longer meeting criteria for SAA without transfusions and G-CSF, and no response (NR) was classified as still meeting criteria for SAA or continuous transfusion dependency.

### 2.3. Statistical Analysis

Data are presented as mean ± SD. Statistical analysis was performed using the chi-square test, followed by the *t*-test upon Gaussian distribution and Wilcoxon signed-rank test upon non-Gaussian distribution. Kaplan-Meier curves were used to estimate survival outcomes. A *P* value < 0.05 was considered statistically significant. All statistical analyses were performed using the SPSS 17.0 statistical package.

## 3. Results

### 3.1. Patients' Characteristics

A total of 88 SAA patients were treated with IST, including 40 recipients of combined therapy with rhTPO and 48 controls without rhTPO administration. There was no statistical significance with regard to demographic or clinical characteristics between patients treated with and without rhTPO (Table 1). The median follow-up was 27.5 months (range of 6.0 to 52.0 months) for all patients, 36.5 months (range of 6 to 52.0 months) for patients treated with rhTPO, and 18.0 months (range of 6 to 48.0 months) for those without rhTPO administration, respectively.

### 3.2. Hematologic Response

The hematologic response rates of all 88 patients were 31.8% at 3 months, 51.1% at 6 months, and 56.8% at 9 months, respectively. Outcomes of the 40 recipients of rhTPO treatment achieving hematologic responses at 3, 6, and 9 months were 17 (42.5%, 3 CR + 14 PR), 25 (62.5%, 8 CR + 17 PR), and 27 (67.5%, 10 CR + 17 PR), respectively. Of the 48 patients with no rhTPO treatment, the hematologic response rates were 22.9% (11/48), 41.6% (20/48), and 47.9% (23/48) at 3, 6, and 9 months, respectively. The difference of the response rates of patients treated with and without rhTPO tended to be statistically significant at 3, 6, and 9 months (*P* = 0.0665, *P* = 0.0579, and *P* = 0.0847, resp.) (Table 2).

### 3.3. Megakaryocyte Recovery in Bone Marrow

**Figure 1:**
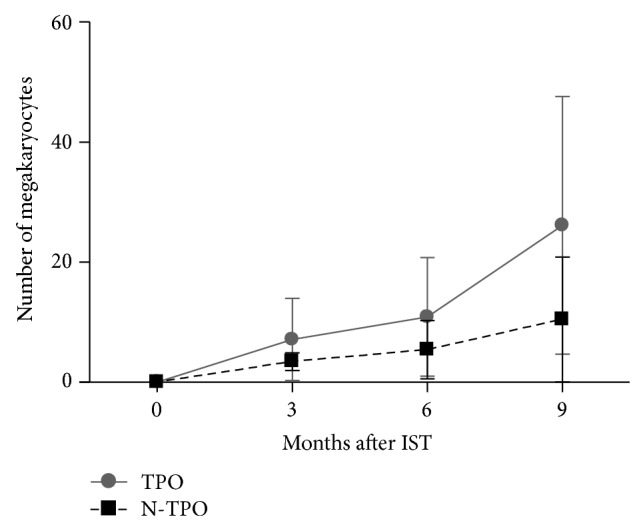
Bone marrow megakaryocytes in patients treated with and without rhTPO at 3, 6, and 9 months (all *P* < 0.05).

In patients treated with rhTPO, megakaryocyte counts (according to bone marrow biopsies of 1 cm × 1 cm) were 9 (*n* = 32, range 0–35) at 3 months, 13 (*n* = 31, range 0–41) at 6 months, and 32 (*n* = 29, range 0–79) at 9 months. The number of megakaryocytes in patients without rhTPO treatment was 3 (*n* = 37, range 0–5), 5 (*n* = 34, range 0–19), and 9 (*n* = 32, range 0–42) at 3, 6, and 9 months, respectively. In our study, there was a significant increase of megakaryocytes in patients treated with rhTPO at 3, 6, and 9 months (*P* = 0.025, *P* = 0.021, and *P* = 0.011, resp.) (Table 3, Figure 1).

### 3.4. Platelet Transfusion Independence

Platelet transfusion independence was achieved in 28/40 (70%) patients treated with rhTPO in comparison with 25/48 (52%) patients without rhTPO administration in one year. No statistical significant association was found between the two groups (*P* = 0.126). The median time to platelet transfusion independence in patients treated with rhTPO was 109 days (range, 32–285) after IST and 156 (range, 47–336) in patients without rhTPO administration, showing a statistically significant correlation (*P* = 0.029).

### 3.5. Recovery of Reticulocytes

The median circulating reticulocyte count of patients treated with and without rhTPO administration was 57 × 10^9^/L, 65 × 10^9^/L, and 82 × 10^9^/L and 42 × 10^9^/L, 61 × 10^9^/L, and 79 × 10^9^/L at 3, 6, and 9 months, respectively. The circulating reticulocyte count tended to be statistically significant in patients treated with rhTPO when compared to those without rhTPO treatment at 3 months (*P* = 0.0662) but showed no statistical association between the two groups at 6 and 9 months (*P* = 0.7022 and *P* = 0.8467, resp.). On the other hand, the median percentage of circulating reticulocytes in patients treated with or without rhTPO at 3, 6, and 9 months was 1.5%, 2.2%, and 1.9% and 0.9%, 1.9%, and 1.7%, respectively. In this case, the percentage of circulating reticulocytes was significantly higher in patients treated with rhTPO when compared to those without rhTPO treatment at 3 months (*P* = 0.025). The two groups at 6 and 9 months presented no statistical association (*P* = 0.486, *P* = 0.643, resp.) (Table 4).

### 3.6. Red Blood Cell Transfusion Independence

Red blood cell (RBC) transfusion independence was achieved in 29/40 (72.5%) patients treated with rhTPO and 28/48 (58.3%) patients without rhTPO administration in 6 months (*P* = 0.032). The median time to RBC transfusion independence in patients treated with and without rhTPO was 83 days (range, 29–264 days) and 131 days (range, 37–296 days) after IST, respectively. There was no statistically significant difference in response to treatment between the two groups (*P* = 0.185).

### 3.7. Survival Rate

**Figure 2:**
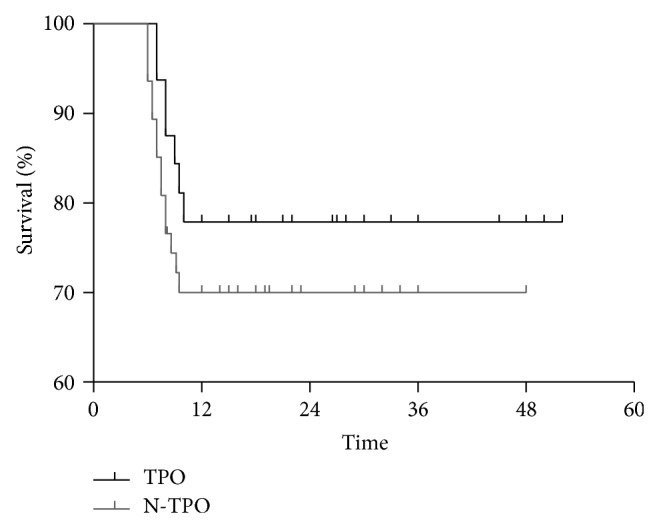
Overall survival rate of patients treated with and without rhTPO. The log-rank test shows that the survival rate of the two groups presented no statistical significance (*P* = 0.394).

The overall survival (OS) rate of patients treated with and without rhTPO was 78.1% and 70.2%, respectively. The log-rank test showed no statistical significance between OS rates of the two groups (*P* = 0.394) (Figure 2).

### 3.8. Clonal Evolution

In this cohort, 2/40 (5%) patients treated with rhTPO and 1/48 (2.1%) nontreated patients progressed to MDS (*P* = 0.588). Cytogenetic abnormalities identified by fluorescence* in situ* hybridization indicated that all three patients presented chromosome 7 deletion.

In order to evaluate whether rhTPO administration caused bone marrow fibrosis, 31 patients treated with rhTPO were subjected to bone marrow biopsy examination. No fibrosis or reticulin proliferation was observed in bone marrow biopsy specimens among all 31 subjects at 3, 6, and 9 months. In these groups of patients, the reticulin score was 0 (28 cases) or 1+ (3 cases), according to the standard scoring system (scores 3+ and 4+ defined as apparent myelofibrosis). Only one patient's reticulin score increased to 2+ when she developed MDS at 17 months.

## 4. Discussion

SAA is a bone marrow failure disorder characterized by pancytopenia, caused often by an autoimmune attack on the bone marrow. The hematologic response rate is 60–75% after receiving IST [9], but successful bone marrow recovery depends on recovery of the immune and hematopoietic function of the residual hematopoietic stem cells. Moreover, frequent blood transfusions during treatment can lead to many complications, including iron overload, transfusion-related infection, and even ineffective transfusion [7].

TPO stimulates the proliferation, differentiation, and maturation of megakaryocytes through the hematopoietic cytokine receptor c-MPL [3, 10]. c-MPL contains two cytokine receptor homology modules (CRMs) and is expressed primarily on hematopoietic cells, particularly in megakaryocytes and promegakaryocytes [11]. The binding of TPO to CRM1 promotes proliferation and differentiation of megakaryocytes [12] via the JAK2/STAT5 and MAPK pathways [13].

The level of TPO expression differs among different types of thrombocytopenia. In immune thrombocytopenic purpura (ITP), TPO levels and platelet production are normal since thrombocytopenia is mainly caused by autoantibody destruction. In congenital amegakaryocytic thrombocytopenia (CAMT), there is a compensatory increase of TPO levels as a result of a defective c-MPL expression [14]. In thrombocytopenia associated with acute leukemia, low TPO levels are presumably caused by the presence of TPO receptor on myeloid leukemic cells. In patients receiving chemotherapy, TPO levels are increased as a result of decreased c-MPL expression [15]. In patients with AA, TPO levels are significantly elevated when compared to those in ITP [16].

Despite the fact that AA patients present elevated TPO levels, Olnes et al. [7] reported that eltrombopag, a TPO peptide mimetic, improved multilineage clinical responses in refractory SAA patients. In that study, 44% of patients achieved a favorable hematologic response in at least one lineage. Furthermore, 36% of patients no longer required platelet transfusion after eltrombopag treatment. The majority of responders that achieved platelet transfusion independence had an average increase in platelet count of 44 × 10^9^/L. Interestingly, erythroid and neutrophilic lineages increased significantly in 6 and 9 patients, respectively. These clinical results support the hypothesis that TPO/c-MPL signaling can directly improve trilineage hematopoiesis.

Recently, Desmond et al. [17] reported the long-term safety and efficacy of eltrombopag in 43 patients with refractory SAA. The overall response rate was 40% at 3-4 months, including tri- and bilineage responses. Five patients with near-normalization of blood counts had drug discontinued at a median of 28.5 months, and all maintained stable counts for a median of 13 months. Eight patients developed new cytogenetic abnormalities on eltrombopag treatment, including 5 subjects with loss or partial deletion of chromosome 7.

In the present study, the hematologic response in at least one lineage presented earlier in SAA patients treated with rhTPO when compared to those not receiving rhTPO. The patients receiving rhTPO achieved an increase in megakaryocyte counts, a decrease in platelet transfusion, and an increased rate of platelet transfusion independence. These results suggested that rhTPO treatment could recover megakaryocytopoiesis and platelet production.

TPO exerts its effects on both hematopoietic stem cells and progenitor cells [11, 18]. CAMT presents initially with thrombocytopenia that subsequently progresses into pancytopenia, suggesting that c-MPL is expressed on multiple hematopoietic lineages [14]. The expression of c-MPL in megakaryocyte-erythrocyte progenitors could facilitate both megakaryocytopoiesis and erythropoiesis [19]. TPO has effects on burst-forming unit-erythroid colonies* in vitro* [20]. Olnes et al. [7] observed clinically significant responses in erythroid, neutrophil, and platelet lineages, with normalization of bone marrow cellularity and trilineage hematopoiesis after eltrombopag treatment.

Our study showed that rhTPO treatment improved erythropoiesis. Circulating reticulocytes were elevated in patients treated with rhTPO at 3 months. The time to RBC transfusion independence was also diminished in rhTPO-treated patients at 6 months. These results suggested that rhTPO administration could accelerate erythropoiesis recovery.

Komatsu et al. administered rhTPO to AA or MDS patients. Their results indicated that rhTPO administration can increase platelet count, achieving multilineage response in some patients [21].

It has been demonstrated that rhTPO treatment can increase MDS transformation and the degree of fibrosis. Our study showed that 2 patients treated with rhTPO and one nontreated patient progressed to MDS, showing no statistical significance between groups. All 3 patients presented chromosome 7 abnormalities. One patient developed bone fibrosis when she progressed to MDS at 17 months. These results suggest that there is no significant association between rhTPO administration and an increased risk of transformation to MDS or bone fibrosis, but this hypothesis needs to be further elucidated.

In our study, OS rate was not found to be statistically significant between the two groups. Nevertheless, our results suggest that rhTPO treatment can improve megakaryocytopoiesis recovery, reducing the frequency of transfusions and the hospitalization time in SAA patients receiving IST. Additionally, transfusion-related complications, such as antibody production or infections, might be reduced.

In summary, our results indicated that rhTPO administration improved hematologic response and bone marrow recovery and reduced the need for transfusion in SAA patients receiving IST. These results suggest a potential therapeutic role of rhTPO as adjuvant therapy in the treatment of SAA.

## Figures and Tables

**Table 1:** Patients' characteristics.

	With rhTPO	Without rhTPO	*P* value
Patients number	40	48	
Median age (year, range)	36 (16–66)	32 (16–68)	0.74
Gender, male/female	18/22	27/21	0.39
Severity of disease			0.51
VSAA	27	28	
SAA	13	20	
Neutrophil counts, ×10^9^/L (median, range)	0.15 (0–1.31)	0.16 (0–1.47)	0.46
Platelet counts, ×10^9^/L (median, range)	9 (0–28)	10 (0–34)	0.75
Reticulocyte counts, ×10^9^/L (median, range)	18 (0.3–43)	14 (0.4–50)	0.62
Lymphocyte counts, ×10^9^/L (median, range)	1.48 (0.41–4.17)	1.52 (0.39–3.99)	0.87
Interval from diagnosis to ATG (median, range), days	19 (1–416)	21 (1–839)	0.58

**Table 2:** Hematological response to patients treated with and without rhTPO.

Time	Response	Patients with rhTPO, number (%)	Patients without rhTPO, number (%)	*P* value
At 3 months	Total	**17 (42.5%)**	**11 (22.9%)**	0.0665
	CR	3 (7.5%)	1 (2.1%)	
	PR	14 (35%)	10 (20.8)	

At 6 months	Total	**25 (62.5%)**	**20 (41.6%)**	0.0579
	CR	8 (20%)	5 (10.4%)	
	PR	17 (42.5%)	15 (31.2%)	

At 9 months	Total	**27 (67.5%)**	**23 (47.9%)**	0.0847
	CR	10 (25%)	7 (14.6%)	
	PR	17 (42.5%)	16 (33.3%)	

CR: complete response; PR: partial response.

**Table 3:** Recovery of bone marrow megakaryocytes in patients treated with and without rhTPO.

Time	Patients with rhTPO	Patients without rhTPO	*P *value
At 3 months	*n* = 32	*n* = 37	
Megakaryocytes	9 (0–35)	3 (0–5)	0.025^*^
At 6 months	*n* = 31	*n* = 34	
Megakaryocytes	13 (0–41)	5 (0–19)	0.021^*^
At 9 months	*n* = 29	*n* = 32	
Megakaryocytes	32 (0–79)	9 (0–42)	0.011^*^

^*^ *P* < 0.05.

**Table 4:** Circulating reticulocyte recovery in patients treated with and without rhTPO.

Time	Reticulocyte counts (×10^9^/L)		*P* value	Reticulocyte percentage (%)		*P* value
	With rhTPO	Without rhTPO		With rhTPO	Without rhTPO	
At 3 months	57 (21–98)	42 (13–76)	0.0662	1.5 (0.2–2.9)	0.9 (0.3–1.4)	0.025^*^
At 6 months	65 (28–106)	61 (22–116)	0.7022	2.2 (0.2–3.8)	1.9 (0.3–3.5)	0.486
At 9 months	82 (25–149)	79 (35–127)	0.8467	1.9 (0.2–3.6)	1.7 (0.2–3.5)	0.643

^*^ *P* < 0.05.
